# Extended infusion of rituximab combined with steroids is effective in inducing remission and reducing relapse in adult minimal change disease

**DOI:** 10.1186/s12882-021-02437-4

**Published:** 2021-07-01

**Authors:** Diankun Liu, Zhanmei Zhou, Mengyi Wang, Sheng Nie, Jun Li, Bianxiang Hu, Wenjuan He, Guobao Wang, Jun Ai

**Affiliations:** 1 National Clinical Research Center for Kidney Disease, Guangdong Provincial Clinical Research Center for Kidney Disease, Nanfang Hospital, Southern Medical University, Guangzhou, China; 2 Renal Division, Nanfang Hospital, Southern Medical University, 1838 North Guangzhou Ave, Guangzhou, 510515 China

**Keywords:** Rituximab, Minimal change disease, Adult, Steroids

## Abstract

**Background:**

Minimal change disease is a common cause of nephrotic syndrome in adults. Higher relapse rate put patients at risk of steroids toxicity due to long-term exposure. Rituximab has been suggested to maintain long time remission and withdraw steroids and other immunosuppressants with fewer adverse events. However, optimal dose and dosing interval have not been explored.

**Methods:**

Twenty-five patients were enrolled from 2017-10 to 2020-03 in Nanfang Hospital in China. Clinical and biological data were extracted from medical records and laboratory databases. Therapy composed of 375mg/m^2^ rituximab once three weeks for 3 dose and corticosteroid was applied. Complete remission was defined as reduction of proteinuria to 0.3g/d. Remission rate, relapse rate, steroids used before and after rituximab therapy and adverse effects were documented at a mean time of 14.71 months.

**Results:**

Twenty-two patients achieved complete remission for an average of 3.26 months and only 3 patients experienced one relapse respectively during the follow-up period. The mean remission maintenance time was 11.6 months, and was 5 months after steroids withdrawal. Steroids dose at last follow-up was 6.09mg/d, which was significantly reduced compared to 28.15mg/d before rituximab. Relapse rate before and after rituximab was 1.43 and 0.1, respectively. Only four minor adverse events were recorded.

**Conclusions:**

Therapy consisted of 375mg/m^2^ rituximab once three weeks for 3 dose combined with corticosteroid is effective in inducing remission in adult patients with minimal change disease. Both of the relapse rate and dose of steroids used are significantly decreased with fewer side effects.

**Supplementary Information:**

The online version contains supplementary material available at 10.1186/s12882-021-02437-4.

## Background

Minimal change disease (MCD) is a major cause of nephrotic syndrome (NS), presented as heavy proteinuria and hypoalbuminemia. It is diagnosed by the absence of glomerular lesions on light microscopy, negative immunofluorescence and extensive podocyte foot process effacement on electron microscopy in renal biopsy. MCD contributes to 70-90% of children > 1 year of age with idiopathic NS. While the proportions, decreasing with age, is about 10-15% in adult patients [1–3]. Corticosteroids including prednisone and methylprednisolone are the primary treatment regimen used at disease onset. Although most of the adult patients respond well to steroid treatment and the long-term prognosis is excellent with remission rate of 75-95% [4], longer remission time, higher tendency to relapse and higher incidence of acute kidney injury when compared with children, as well as no consensus on the optimal dose and duration of therapy, put adults at risk of prolonged exposure of steroids and other immunosuppressive agents [5, 6].

Rituximab (RTX) is a chimeric monoclonal antibody, inhibiting CD20-mediated B-cell proliferation and differentiation, which was originally developed to treat B cell non-Hodgkin’s lymphoma [7]. It was firstly introduced to treat steroid-dependent nephrotic syndrome (SDNS) in 2004, when a child diagnosed with idiopathic thrombocytopenic purpura (ITP) and NS received RTX therapy and achieved remission [8]. Since then, more and more studies have proven the efficacy of RTX use in MCD about inducing remission, reducing relapse rate, providing long-term remission off steroids or other immunomodulatory drugs [9–17]. RTX also showed a favorable long-term safety profile compared to traditional therapy, with lower incidence of infection, no risk of metabolic disorder such as glucose intolerance or osteoporosis, and no impact on the reproductive system. The major side effects consisted of infusion-related reaction, HBV reactivation in HBsAg-negative/HBcAb-positive patients and rarely pneumocystis jirovecii pneumonia (PCP) and progressive multifocal leukoencephalopathy (PML) [18].

However, most of the investigation focused on MCD in children. There are only a few retrospective, observational studies regarding RTX use in adult patients [19–30]. In these limited literatures, initial RTX dosage is quite different not only between researches but also in the same article, which makes comparison between different therapeutic strategies impossible. Moreover, some studies have implicated that the serum level of RTX in patients with nephrotic syndrome is lower than that of patients in other disease like rheumatoid arthritis (RA) [31] probably due to the loss of RTX in the urine. Thus, it is crucial to find the optimal dose of RTX in adult MCD, which combines the maximum remission duration with minimal side effects.

In the current analysis, we aim to evaluate the efficacy of a regimen of 3 applications of 375mg/m^2^ dose of RTX three weeks apart together with steroids in adult patients with biopsy-proven MCD, and to show the relapse rate and discontinuation or tapering of steroids during the follow-up period after RTX therapy.

## Methods

### Study population and clinical characteristics

Twenty-five adult patients (age > 18 years old) with biopsy-proven MCD were recruited in this study from 2017-10 to 2020-03 at the Department of Nephrology at Nanfang Hospital in China. One patient was excluded because of exacerbation of pruritus after first infusion of RTX. In the remaining 24 patients, previous medical history of every participant had been reviewed to define the clinical subtype of MCD: steroid-dependent nephrotic syndrome (SDNS), steroid-resistant nephrotic syndrome (SRNS), infrequently relapsing nephrotic syndrome (IRNS) and frequently relapsing nephrotic syndrome (FRNS). SDNS was defined as relapse during steroid therapy or with 15 days of discontinuation. SRNS was defined as no response to prednisone 1mg/kg per day or 2mg/kg every other day within 16 weeks. Infrequently relapsing nephrotic syndrome was defined as < 2 relapses per 6 months or < 4 relapses per 12 months. Frequently relapsing nephrotic syndrome was defined as ≥ 2 relapses per 6 months or ≥ 4 relapses per 12 months [3, 32].

We also obtained age at disease onset, previous immunosuppressive therapy, disease duration, relapse rate from the medical history. Clinical data were obtained from the medical records at admission, which consisted of age at first RTX infusion, sex, comorbidities, current immunosuppressive regimen, the dosage of steroids before RTX therapy and at last follow-up. Biological data were obtained from the laboratory databases, including 24h urine protein excretion, serum albumin, absolute count of CD19+ B cell, serum creatinine and estimated glomerular filtration rate (eGFR) estimated with Modification of Diet in Renal Disease (MDRD) equation at the time of RTX initiation, 3, 6, 9, 12, 20, 32, 44, 56 weeks after the first infusion of RTX therapy and at last follow-up.

The study protocol was approved by the medical ethics committee of Nanfang Hospital, Southern Medical University and is in accordance with the Declaration of Helsinki.

### Rituximab therapy

Patients received 375mg/m^2^ RTX treatment once three weeks for 3 dose and corticosteroids which dose is maintained according to the last therapy. Before infusion, loratadine was prescribed to lower the incidence of infusion-related reaction. Infusion rate of RTX started at 20ml/hour, which was doubled after 30 minutes of infusion if no infusion-related reaction happened, until reaching the highest rate of 120ml/hour. Electrocardiogram monitor was used during the infusion period to check the vital sign of patients. Patients were all treated with trimethoprim-sulfamethoxazole (TMP-SMX) for a week after infusion of RTX to prevent PCP.

Remission was defined according to 2012 KDIGO (Kidney Disease Improving Global Outcomes) Clinical Practice Guideline for Glomerulonephritis [32]. Complete remission (CR) was defined as reduction of proteinuria to 0.3g/d, normal serum creatinine and serum albumin > 35g/L; Partial remission (PR) was defined as reduction of proteinuria to 0.3-3.5g/d and a decrease > 50% from baseline, and stable serum creatinine (change in serum creatinine < 25%). Relapse was defined as proteinuria > 3.5g/d after complete remission has been obtained.

### Statistical analyses

All study data were stored in a standard Excel database. Statistical analysis was performed using SPSS version 20.0 for Windows (SPSS Inc., Chicago, IL, USA), GraphPad version 8.3.0 (GraphPad Software, San Diego, CA) and STATA version 15.0 for Mac (StataCorp LLC, Texas, USA). All continuous variables were expressed as mean or mean ± standard deviation (SD). Categorical variables were presented as frequencies and percentages [n(%)]. Differences between relapse and dosage of steroids before and after RTX therapy were analyzed by t-test for continuous variables and chi-square test or Fisher exact tests, if appropriate for categorical variables. Kaplan-Meier analysis was used to calculate the cumulative incidence of complete remission. Longitudinal data analyses were performed to illustrate the variation of urinary protein, serum albumin and serum creatinine. The value of *P* < 0.05 was considered statistically significant.

## Results

### Patients characteristics and clinical parameters

We included 24 adult patients from 2017-10 to 2020-03 at the Department of Nephrology at Nanfang Hospital in China. Baseline characteristics of study population were presented in Table 1. The detailed information of each patient was illustrated in Table S1 in Additional file 1. All the patients were male, and were diagnosed as MCD based on clinical manifestation and pathological results from renal biopsy. 9 patients have adult-onset MCD, while 15 patients were recognized as having nephrotic syndrome during childhood. The average age at RTX infusion was 23.58 years old (18-46), and was 18.88 years old (3-46) at disease onset. The disease duration ranged from 0.25 to 14.58 years. All the patients received at least 1 course of corticosteroids or other immunosuppressive therapy, such as Cyclosporin (11), Tacrolimus (12), Cyclophosphamide (1), Mycophenolate mofetil (8), Chlorambucil (4), Leflunomide (1), Tripterygium glycosides (3), with calcineurin inhibitors (CNI) consisting of cyclosporin and tacrolimus being the most common used immunosuppressants. Only 1 patient (patient 24) avoided steroids treatment because of glaucoma induced by prednisone, and received tacrolimus treatment alone. The remaining 23 patients accepted steroids treatment since disease onset. 17 patients were defined as SDNS, 6 patients were recognized as SRNS. While, 2 patients had FRNS and 21 patients were considered having IRNS.

**Table 1:** Clinical characteristics of study population before rituximab therapy

Characteristics	Patients (*n* = 24)
Age at onset of disease, years	18.88±8.57 (3-46)
Age at first rituximab infusion, years	23.58±7.89 (18-46)
Male gender	24 (100%)
Disease duration, years	4.29 (0.25-14.58)
24h urinary protein excretion, g	9.49±7.55
Serum creatinine, μmol/L	118.58±109.24
eGFR (MDRD), ml/min*1.73m^2^	103.36±43.83
Serum albumin, g/L	22.57±6.94
CD19+ B cell count, n/μL	343.89±269.75
Previous immunosuppressive therapy	62
Long term corticosteroids	22
Cyclosporine	11
Tacrolimus	12
Cyclophosphamide	1
Mycophenolate mofetil	8
Chlorambucil	4
Leflunomide	1
Tripterygium glycosides	3
Prednisone dose before rituximab therapy, mg/day	28.15 (5-50)
Relapse rate before rituximab therapy, number/year	1.43 (0-4.36)
Follow-up time, months	14.71 (8-36)

Quantitative data was expressed as mean or mean ± standard deviation (SD), categorical data was presented as frequencies and percentages [n (%)]

*Abbreviations*: *eGFR* estimated glomerular filtration rate, *MDRD* Modification of Diet in Renal Disease

Nineteen patients presented with nephrotic syndrome at the time of first infusion of RTX. 3 patients having sub-nephrotic range proteinuria and 2 patients in corticosteroids-induced CR were treated in order to improve steroids-related complication or avoid long-term toxicity of corticosteroids through steroids tapering or withdrawal. The average 24-hour urine protein excretion was 9.49g/d (0.07-30.5) and the mean serum albumin was 22.57g/L (13.9-36.8). 6 patients had elevated serum creatinine. The mean eGFR was 103.36ml/min per 1.73m2 (12.1-186.68). Pre-treatment CD19+ B cell count was 344/μL (92-1208) an average. The average follow-up time after first infusion was 14.71 months (8-36).

### Rituximab therapy

Nineteen patients received 375mg/m^2^ RTX treatment once three weeks for 3 dose and corticosteroids which dose is maintained as prescribed previously. Four patients received the fourth dose of RTX, in which 3 patients had overt proteinuria at the beginning and before the third dose of RTX (12.1-24.73). The remaining patient was treated with the fourth dose in consideration of NS refractory to each available therapy for 93 months. Only 1 patient declined the third dose of RTX because of recurrent and uncontrolled onychomycosis.

### Response to rituximab treatment

Twenty-two patients achieved CR for an average of 3.26 months (0.75-11) after the first infusion of RTX with 13 patients achieved CR during the treatment period (Table 2). The longest remission time is 11 months, while the shortest remission time is just before the second dose of RTX infusion. Two patients showed no response even acquired 4 dose of RTX. The remaining 2 patients being in remission before treatment were in maintained remission at last follow-up despite of steroids withdrawal. In 2 patients with no response (NR), 1 patient were treated with prednisone (1mg/kg) and achieved CR thereafter and 1 patient showed remarkable decrease in proteinuria from 30.5g/d initially to 7.62g/d and normal serum albumin level at last follow-up.

**Table 2:** Tapering doses of prednisone and relapse rate after rituximab therapy

Prednisone dose at last follow up, mg/day	6.09±7.38 (0-25)
Duration of prednisone withdrawal, months	12.64 (6-25)
Remission time, weeks	13 (3-44)
Remission time, months	3.26 (0.75-11)
Remission maintenance time, months	11.6 (0.25-31)
Remission maintenance time after withdrawal of prednisone, months	5.0 (1-11)
Relapse rate after rituximab therapy, number/year	0.1 (0-0.85)

Variables were expressed as mean or mean ± standard deviation (SD)

**Fig. 1:**
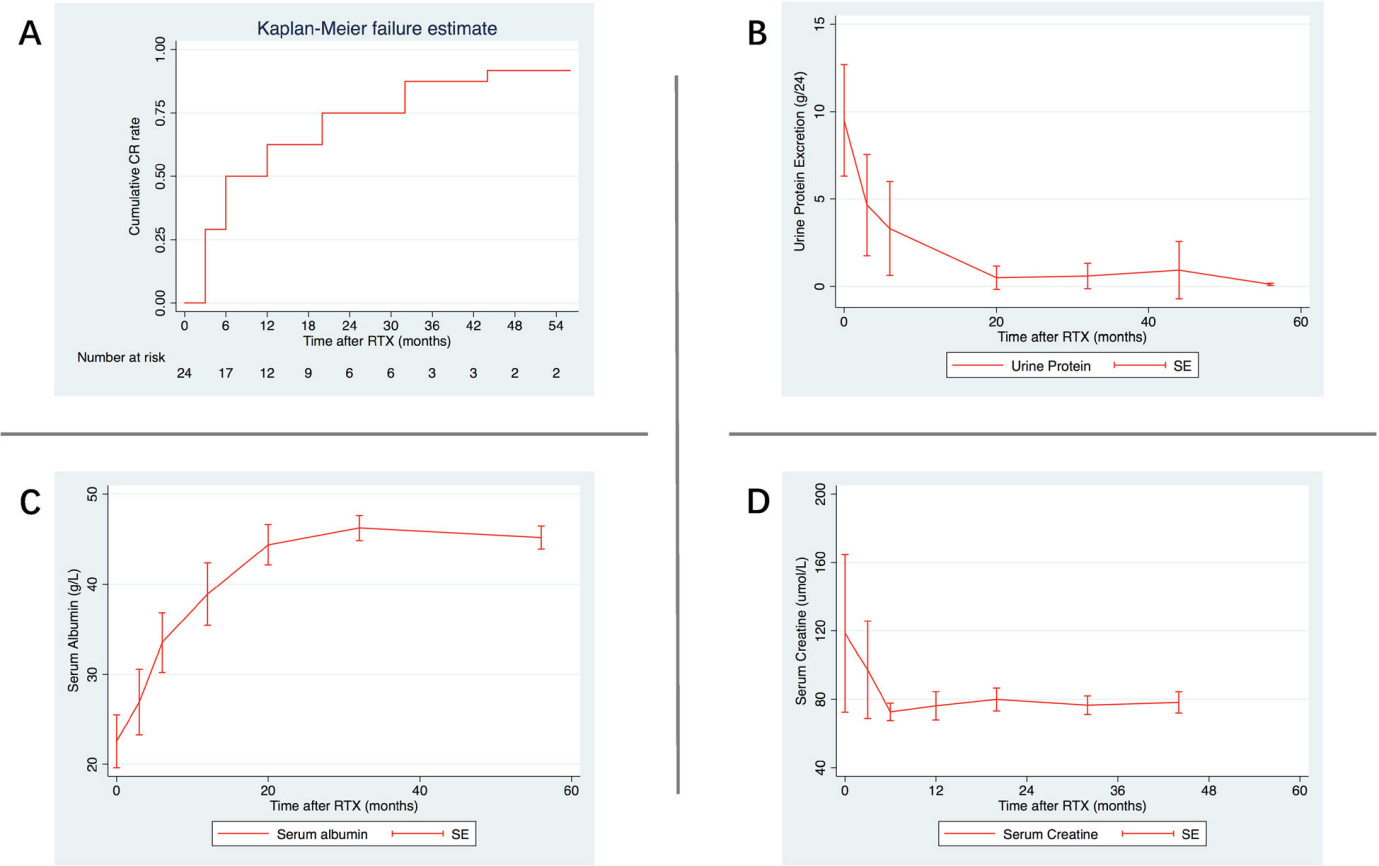
**A** The cumulative incidence of complete remission after rituximab therapy during a 56-week follow up; **B** The variation of urine protein excretion after rituximab therapy; **C** The variation of serum albumin after rituximab therapy; **D** The variation of serum creatinine after rituximab therapy. Abbreviations: RTX, rituximab

Cumulative incidence of remission, and changes of 24-hour urinary protein excretion, serum albumin and serum creatinine were illustrated in Table 3, Fig. 1 and Figure S1. The cumulative response rate was 91.67% (22/24), and all the patients with response to RTX therapy achieved CR. The response rate showed no differences between childhood-onset and adult-onset patients, which were showed in Tables S2 and S3.

**Table 3:** Change of proteinuria, serum creatinine, and serum albumin after rituximab treatment

	0W	3W	6W	12W	20W	32W	44W	56W	LFU
Proteinuria, g/24h	9.49	4.65	3.31	1.05	0.49	0.59	0.93	0.12	1.48
Serum Creatinine, μmol/L	118.58	97.25	72.65	76.2	79.93	76.54	78.17	79.5	77
Serum Albumin, g/L	22.57	26.92	33.52	38.9	44.39	46.27	42.85	45.2	40.44
CD19+ B cell count, n/μL	344	6	4	/	/	/	/	/	/

*Abbreviations*: *W* week, *LFU* last follow up

### Tapering doses of steroids and relapse after RTX

**Fig. 2:**
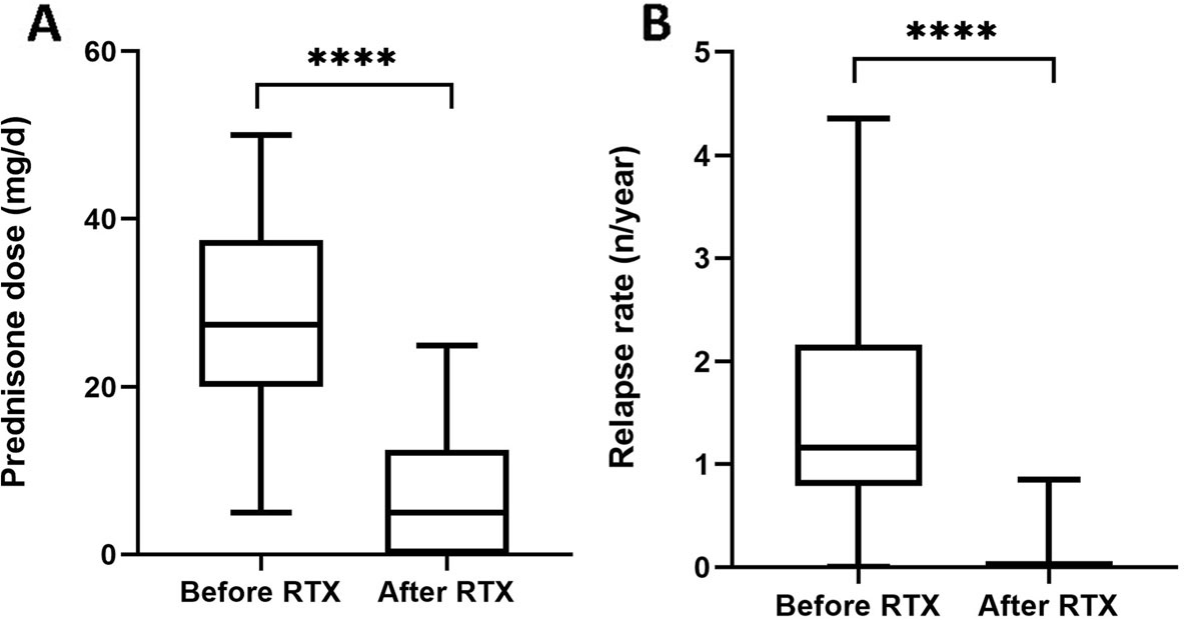
**A** Prednisone dose is significantly different in patients before and after rituximab treatment; **B**: Relapse rate is significantly different in patients before and after rituximab treatment. Abbreviations: RTX, rituximab. *P* value is calculated using paired t-test to demonstrate the differences in prednisone dosage and relapse rate between the two groups. **** *P* < 0.0001

Steroids tapering protocol was detailed in Additional file 2. Tapering of steroids therapy, remission maintenance time with or without steroids were illustrated in Table 2 and Table S4. Twenty-one patients experienced relapse at the dose of steroids before the first infusion of RTX. At last follow-up, the mean prednisone dose is 6.09mg/d (0-25), which is significantly reduced compared to 28.15mg/d (5-50) at the beginning of RTX therapy, which was illustrated in Fig. 2. Eleven patients were out of steroids therapy and maintained in remission during the follow-up time. The mean remission maintenance time is 11.6 months (0.25-31) in total and 5 months (1-11) after corticosteroids withdrawal.

In 22 patients in remission after RTX therapy, 3 patients experienced relapse during steroids tapering. One patient (Patient 5) received additional dose of RTX and achieved remission rapidly. One patient (Patient 16) also achieved remission with increased prednisone dosage. The remaining patient (patient 11) experienced relapse after maintaining remission for 16 months, and was treated with the fourth dose of RTX. However, response condition was not recorded because that relapse time is near the time of last follow-up. In 3 patients with relapse, circulating CD19+ B cell count were 33/μL (patient 5), 459/μL (patient 11), and 2/μL (patient 16), respectively. The average relapse rate after RTX was significantly lower than that before (0.1% vs 1.43%), which was depicted in Fig. 2.

When dividing patients into childhood-onset and adult-onset MCD, both the steroids dosage and the relapse rate showed no significant differences before and after rituximab therapy (Table S2 and S3).

### Adverse effects

During the treatment period and follow-up time, only four minor adverse events were recognized (Table 4). One patient experienced exacerbation of pruritus, which was presented before RTX treatment, and discontinued the following infusion. Two patients in the cohort experienced infusion-related reaction. One had allergic reaction presenting as urticaria and topical pruritus at first infusion, which is alleviated by temporary stop the infusion of RTX and use of anti-histamine agents. Another patient showed flu-like symptoms. The remaining patient refused the third dose of RTX because of uncontrolled onychomycosis, which already existed before RTX therapy, probably due to previous long-term use of steroids and other immunosuppressive drugs.

**Table 4:** Side effects of rituximab during follow-up

Pruritus	1
Infusion-related reaction	2
Allergic reaction	1
Flu-like symptoms	1
Skin and soft tissue infection	1

## Discussion

In this case series, we investigated the efficacy of rituximab combined with corticosteroids in inducing remission in adult patients with either SDNS or SRNS of MCD. To our knowledge, it is the first retrospective observational study that apply 375mg/m^2^ RTX infusion once three weeks for 3 dose together with steroids in adult patients with biopsy-proven MCD in China. In our research, 91.67% patients achieved CR in a mean time of 3.26 months (0.75-11), and only three patients experienced one relapse during the follow-up period. The average dose of steroids at last follow-up was significantly lower than that used before rituximab (28.15mg/d versus 6.09mg/d).

MCD is a common cause of nephrotic syndrome in children, which was considered equal to steroid-sensitive nephrotic syndrome when renal biopsy was not performed. While in adult, MCD accounted for about 15% of idiopathic nephrotic syndrome in previous research [3], and 30% of that in China [33]. The pathological hallmark of MCD is absence of visible alteration by light microscopy and podocyte foot process effacement. Although patients diagnosed as MCD responded well to corticosteroids, longer time to remission, higher relapse rate and higher incidence of AKI in adults put them at risk of steroids and other immunosuppressive drug toxicity.

RTX has been proven to be effective in inducing remission of MCD. However, most of the researches were restricted in children. There are only a few small-sampled observational studies regarding RTX use in adult patients. The reported incidence of CR of MCD after RTX therapy in adult patients ranged from 50% to 100% [19–30]. However, it is difficult to draw any reliable conclusion from previous studies. Firstly, different therapeutic protocols of RTX were used not only between different researches, but also in the same study [6, 25–27]. Moreover, in certain researches, patients were already in remission due to previous treatment [19, 27, 29]. Adult patients with biopsy-proven focal segmental glomerulonephritis (FSGS) were enrolled in certain studies [20, 26–28, 30]. Last but not least is that different immunosuppressive drugs excluding steroids were used in the RTX protocols [20, 21, 25–28]. B-cell driven therapy had been used in some researches, which showed promising effects [27, 30]. However, interaction between RTX and podocyte function was also considered important of RTX therapy in MCD [34]. Some studies implied that relapse did not always happen during B cell reconstruction as well [25, 35]. In our study, CD19+ B-lymphocyte cell count at the time of relapse in 3 patients varied from 2-459/μL, which also indicated that effectiveness of RTX in MCD might not be only attributed to B cell depletion. While, we firstly apply 375mg/m^2^ RTX treatment once three weeks for 3 dose combined with corticosteroids in all the patients. Only 4 patients required additional infusion, and 1 patient declined the third dose of RTX resulting from uncontrolled concurrent infection. The incidence of CR in our study is 91.67%, which concluded that RTX combined with steroids is effective in inducing remission in adult MCD patients with considerable remission rate.

The optimal dose of RTX, which combines the maximum remission duration with minimal side effects, is difficult to define, even in lymphoma and rheumatoid arthritis. In lymphoma, R-CHOP-21 schedule provided a longer exposure and maximized the exposure to clinical relevant concentrations of RTX, compared to R-CHOP-14 [36]. While R-CHOP-14 protocol ensured higher C-max and C-min during infusion period. Moreover, in patients manifesting nephrotic syndrome, serum RTX concentration is significantly lower than that in RA [31] or Myasthenia gravis [37]. Probably due to the loss of RTX in urine in patients with remarkable proteinuria. However, loss of RTX in urine was not documented in MCD [38]. Therefore, further investigation with respect to serum concentration of RTX in MCD and superior protocol with optimal initial dose and maintenance dose are required.

Previous researches have suggested RTX therapy was able to reduce the relapses per year and to enable discontinuation or tapering of steroids or other immunosuppressive agents, which is consistent in our study. Relapses per year and dose of steroids used in remission after RTX were considerably reduced compared to those before RTX therapy. The relapse rate is 13.64%, only 3 patients experienced one relapse during follow-up period respectively. In previous literatures, relapse rate varied from 0 to 56% [19–30]. Firstly, the time of follow-up seemed to be the reasonable factor. One study concluded that the median time to relapse is 18 months [25]. Meanwhile, patients showed relapse mostly after 6 months of RTX therapy [19, 21, 24, 25, 27–30]. Therefore, studies conducted shorter follow-up time may not reflect the true relapse rate. Secondly, populations varied across studies. Patients with treatment-naive MCD showed no relapse after RTX therapy [23]. While researches recruiting patients with FSGS demonstrated higher relapse rate [20, 27, 30]. Thirdly, different treatment strategies, either alone or combined with other immunomodulatory drugs may play an important role explaining the inconsistency between literatures. While *Takei* delineated that B cell reconstruction could not be a reliable parameter to indicate the relapse [21] and the effectiveness of RTX in MCD might result from its impact on podocyte function [39], we proposed not to adjust RTX infusion based on the circulating CD-19 positive B-lymphocyte cells, in order to avoid unnecessary exposure of RTX.

Furthermore, the mean dose of steroids used at last follow up is significantly reduced compared to that before RTX therapy, which is in accordance with other reported results [22, 26, 27].

Similar to previous researches, RTX use in adult patients with MCD was quite tolerable. Only 4 adverse events were recorded during the treatment and follow-up period. Infusion-related reaction was considered the most common side effect of RTX, which accounted for 50% of adverse events in our study. One infection presenting as onychomycosis, happened before RTX probably due to long-term use of corticosteroids and other immunosuppressive agents, which was refractory to standard antimicrobial treatment during RTX treatment. HBV reactivation was not detected, as well as PCP or PML, which were considered serious side effects, requiring long term monitor.

Podocyte damage was considered to be crucial in the pathogenesis of MCD [40]. Previously, existence of a circulating mediator produced by abnormal T cells was postulated to disrupt the function of podocyte and increase the permeability of glomerular filtration barrier, which resulted in proteinuria [41, 42]. However, in the last decades, especially as the use of RTX in MCD, B lymphocyte cells were thought to play an indispensable role in the mechanize of MCD. T helper 2 (TH2) cell mediated immunity and its related cytokines, such as IL-4 and IL-13 have been verified to cause proteinuria in murine model through inducing foot process effacement [43, 44]. Additionally, IgG-antibody directed against Ubiquitin Carboxyl-Terminal Hydrolase L1 (UCHL1) was shown to cause podocyte detachment and associated with relapses of idiopathic NS in mice [45]. Apart from the direct influence on B cell, RTX has been proven to bind directly to podocyte Sphingomyelin Phosphodiesterase Acid Like 3b (SMPDL3b) in vitro, which demonstrated its antiproteinuric effect independent of B cell depletion [34]. A diversity of indirect effects, including lipid raft modifications, kinase and caspase activation and effects on apoptotic/antiapoptotic molecules appear to play a crucial role for the observed variability in response to RTX treatment [46]. At the same time, kruppel-like factor 15, a zinc-finger transcription factor expressed in human podocytes, contributes to mediate the beneficial effects of glucocorticoid therapy via stabilization of the actin cytoskeleton of podocytes in MCD [47]. While, in our study, only 2 patients showed no response to RTX treatment, in which one patient was considered responder because of decreasing proteinuria and increasing of serum albumin at last follow up. Thus, it is difficult for us to investigate the differences between responders and non-responders, which might contribute to the study on pathogenesis of MCD and role of B cell or RTX in this disease.

The strength of our research is that we firstly introduced 3 dose protocol of rituximab infusion combined with corticosteroids in the treatment of adult MCD and demonstrated promising efficacy in inducing remission, reducing relapse and tapering steroids without severe side effects. The fixed protocol conducted in this series makes results reliable and comparable, which further promote the use and investigation of RTX in MCD patients.

Our study still have some limitations. First of all, sampling bias is inevitable. Each physician in our department selected RTX therapy for adult MCD patients based on their clinical experience. There was no consensus on patient selection. Moreover, the lack of circulating CD19+ B lymphocyte cells measurement made it impossible to reveal the relationship between remission or relapse and lymphocyte. Additionally, follow-up time is too short that the relapse rate might be underestimated, as well as long-term adverse events. Last but not least is that the results of small sample size study will not accurately reflect the actual remission rate. Consequently, investigations including randomized controlled trials for long-term follow up regarding RTX use in adult MCD are required to further illustrate the effectiveness and safety.

## Conclusion

In summary, our study concluded that 375mg/m^2^ RTX treatment once three weeks for 3 dose combined with corticosteroids is effective in inducing remission in adult patients diagnosed as MCD. The remission rate is 91.67%. During the follow-up period, only 3 patients experienced one relapse respectively. The relapse rate is 13.64%. Dosage of steroids and relapses per year are significantly reduced after RTX therapy compared to those before. Tolerance of RTX is excellent with no severe adverse effects documented.

## Supplementary Information

**Additional file 1: Table S1**. Patient characteristics before rituximab therapy. Illustrating the age, immunosuppressive therapy, relapse rate, serum creatinine, serum albumin, urinary protein excretion, CD19+ B cell count of each patient before rituximab therapy. Showing rituximab courses and prednisone dosage of each participant receiving this therapy. **Table S2**. Baseline characteristics and outcome of rituximab therapy between patients with childhood-onset (<12y) and adult-onset MCD. Comparing the differences in the age at first rituximab infusion, 24h urinary protein excretion, serum creatinine, eGFR, serum albumin, CD19+ B cell count, response rate, prednisone dose before rituximab and at last follow up, relapse rate before and after rituximab between patients with childhood onset MCD (<12y) and patients with adult onset MCD (≥12y). **Table S3**. Baseline characteristics and outcome of rituximab therapy between patients with childhood-onset (<18y) and adult-onset MCD. Comparing the differences in the age at first rituximab infusion, 24h urinary protein excretion, serum creatinine, eGFR, serum albumin, CD19+ B cell count, response rate, prednisone dose before rituximab and at last follow up, relapse rate before and after rituximab between patients with childhood onset MCD (<18y) and patients with adult onset MCD (≥18y). **Table S4**. Change of prednisone dosage after rituximab treatment. Demonstrating the average dose of prednisone at each follow up time after rituximab therapy. **Figure S1**. A: The variation of urine protein excretion of each patient after rituximab therapy; B: The variation of serum albumin of each patient after rituximab therapy; C: The variation of serum creatinine of each patient after rituximab therapy. Abbreviations: RTX, rituximab.

**Additional file 2. **Protocol of steroids tapering in the study.

## Data Availability

Original data was stored in a standard EXCEL database and can be requested via the corresponding author. aij1980@163.com; nfyywanggb@163.com.

## Abbreviations

MCD: Minimal change disease
NS: Nephrotic syndrome
RTX: Rituximab
SDNS: Steroid-dependent nephrotic syndrome
ITP: Idiopathic thrombocytopenic purpura
PCP: Pneumocystis jirovecii pneumonia
PML: Progressive multifocal leukoencephalopathy
RA: Rheumatoid arthritis
SRNS: Steroid-resistant nephrotic syndrome
IRNS: Infrequently relapsing nephrotic syndrome
FRNS: Frequently relapsing nephrotic syndrome
eGFR: Estimated glomerular filtration rate
MDRD: Modification Diet of Renal Disease
KDIGO: Kidney Disease Improving Global Outcomes
TMP-SMX: Trimethoprim-sulfamethoxazole
CR: Complete remission
PR: Partial remission
SD: Standard deviation
CNI: Calcineurin inhibitors
NR: No response
FSGS: Focal segmental glomerulonephritis
TH2: T helper 2
UCHL1: Ubiquitin Carboxyl-Terminal Hydrolase L1
SMPDL3b: Sphingomyelin Phosphodiesterase Acid Like 3b
