# Supplementary material for: Extended infusion of rituximab combined with steroids is effective in inducing remission and reducing relapse in adult minimal change disease
Source: BMC Nephrol. 2021 Jul 1;22:242. doi: 10.1186/s12882-021-02437-4 (PMC8247102; doi:10.1186/s12882-021-02437-4)

**Supplementary materials:**

| **Table S1. Patient characteristics before rituximab therapy** | | | | | | | | | |
| --- | --- | --- | --- | --- | --- | --- | --- | --- | --- |
| Patient number | Immunosuppressive therapy before rituximab | Relapses before rituximab therapy (number/year) | Serum creatinine (μmol/L) | eGFR | Serum albumin (g/L) | 24h urinary protein exretion (g/d) | CD19+ B cell count (n/μL) | Rituximab courses | Prednisone dose (mg/d) |
| 1 | Pred/Pred+CysA/Pred+MMF/Pred+CTX/Pred+FK506/Pred+FK506+Tripterygium glycosides/Pred+Chlorambucil | 1.03 | 68 | 128.96 | 14.8 | 9.4 | / | 4 | 20 |
| 2 | Pred/Pred+MMF | 0.49 | 87 | 88.29 | 34 | 1.48 | / | 3 | 30 |
| 3 | Pred/Pred+FK506 | 0.98 | 79 | 117.77 | 29.5 | 9.17 | / | 3 | 45 |
| 4 | Pred/Pred+CysA/Pred+Chlorambucil | 0.79 | 66 | 131.56 | 24.6 | 6.77 | 219 | 3 | 40 |
| 5 | Pred/Pred+CysA | 4.36 | 204 | 38.58 | 17.6 | 17.53 | 234 | 3 | 20 |
| 6 | Pred/Pred+CysA/Pred+MMF/Pred+Tripterygium glycosides/Pred+FK506/Pred+Chlorambucil | 1.31 | 87 | 104.22 | 18 | 3.4 | 238 | 3 | 30 |
| 7 | Triamcinolone+CysA/Triamcinolone+MMF/Pred+MMF/Pred+CysA | 1.41 | 557 | 12.1 | 13.9 | 21.53 | 238 | 4 | 15 |
| 8 | Pred/Pred+FK506 | 1.50 | 89 | 102.64 | 16.7 | 20.88 | 420 | 3 | 27.5 |
| 9 | Pred/Pred+FK506 | 2.92 | 60 | 156.8 | 17.3 | 10.27 | 222 | 3 | 20 |
| 10 | Pred | 1.09 | 77 | 121.31 | 28.7 | 2.4 | 582 | 3 | 25 |
| 11 | Pred/Pred+FK506 | 0.62 | 53 | 186.68 | 29.1 | 0.07 | 1208 | 3 | 25 |
| 12 | Pred | 0.31 | 76 | 121.81 | 33.8 | 4.03 | 273 | 4 | 5 |
| 13 | Pred/Pred+CysA | 1.23 | 98 | 87.38 | 15.1 | 14.11 | 206 | 3 | 30 |
| 14 | Pred | 2.18 | 65 | 145.9 | 36.8 | 0.27 | 209 | 3 | 50 |
| 15 | Pred/Pred+CysA | 2.18 | 119 | 73.41 | 24.5 | 16.62 | 379 | 3 | 25 |
| 16 | Pred/Pred+Tripterygium glycosides | 0.91 | 79 | 107.67 | 31.4 | 4.9 | 239 | 3 | 12.5 |
| 17 | Pred/Pred+MMF | 2.18 | 205 | 39.19 | 18 | 10.99 | 152 | 3 | 50 |
| 18 | Pred/Pred+CysA/Pred+FK506 | 1.40 | 72 | 125.85 | 24.6 | 5.49 | 142 | 2 | 37.5 |
| 19 | Pred/Pred+CysA/Pred+FK506 | 0.92 | 84 | 101.05 | 19.5 | 8.15 | 335 | 3 | 20 |
| 20 | Pred/Pred+MMF/Pred+CysA/Pred+FK506/Pred+Chlorambucil | 0.79 | 56 | 165.25 | 19.9 | 4.86 | 410 | 3 | 30 |
| 21 | Pred/Pred+Leflunomide/Pred+MMF | 3.00 | 78 | 119.52 | 21.1 | 5.79 | 215 | 3 | 30 |
| 22 | Pred/Pred+FK506 | 2.12 | 118 | 74.12 | 14.8 | 30.5 | 942 | 4 | 40 |
| 23 | Pred | 0.00 | 294 | 21.37 | 17.3 | 12.6 | 268 | 3 | 20 |
| 24 | FK506 | 0.63 | 75 | 109.26 | 20.7 | 6.58 | 92 | 3 | / |
| Abbreviations: Pred, prednisone; CysA, cyclosporin; MMF, mycophenolate mofetil; CTX, cyclophosphamide; FK506: tacrolimus. | | | | | | | | | |

| **Table-S2. Baseline characteristics and outcome of rituximab therapy between patients with childhood-onset (<12y) and adult-onset MCD** | | | |
| --- | --- | --- | --- |
| Characteristics | Patients with childhood onset (n=2) | Patients with adult onset (n=22) | P value |
| Age at first rituximab infusion, years | 18.50±0.71 | 24.05±8.09 | 0.3523 |
| 24h urinary protein excretion, g | 2.05±2.80 | 10.17±7.52 | 0.1492 |
| Serum creatinine, μmol/L | 64.50±16.26 | 123.50±112.90 | 0.4769 |
| eGFR (MDRD), ml/min*1.73m^2^ | 154.20±45.87 | 98.74±41.65 | 0.0863 |
| Serum albumin, g/L | 31.45±3.32 | 21.76±6.64 | 0.0567 |
| CD19+ B cell count, n/μL | 740.50±661.70* | 302.10±192.90 | 0.0245 |
| Response rate | 50.00% | 95.45% | 0.1630 |
| Prednisone dose before rituximab therapy, mg/day | 15.00±14.14 | 29.40±10.87 | 0.0925 |
| Prednisone dose at last follow up, mg/day | 5.00±7.07 | 6.19±7.57 | 0.8332 |
| Relapse rate before rituximab therapy, number/year | 0.47 | 1.52 | 0.1560 |
| Relapse rate after rituximab therapy, number/year | 0.35 | 0.08 | 0.1792 |
| Quantitative data was expressed as mean or mean ± standard deviation (SD), categorical data was presented as frequencies and percentages [n (%)]. Differences between catogorical data were analyzed by Fisher exact tests. Differences between quantitative data were analyzed by t-test. Abbreviations: eGFR, estimated glomerular filtration rate; MDRD, Modification of Diet in Renal Disease. *P < 0.05 for comparison of childhood onset group versus adult onset group. | | | |

| **Table-S3. Baseline characteristics and outcome of rituximab therapy between patients with childhood-onset (<18y) and adult-onset MCD** | | | |
| --- | --- | --- | --- |
| Characteristics | Patients with childhood onset (n=15) | Patients with adult onset (n=9) | P value |
| Age at first rituximab infusion, years | 19.20±1.82* | 30.89±8.77 | <0.0001 |
| 24h urinary protein excretion, g | 9.75±8.91 | 9.06±4.96 | 0.8330 |
| Serum creatinine, μmol/L | 119.40±126.80 | 117.20±78.63 | 0.9635 |
| eGFR (MDRD), ml/min*1.73m^2^ | 111.10±46.58 | 90.46±37.77 | 0.2733 |
| Serum albumin, g/L | 23.11±7.12 | 21.67±6.96 | 0.6317 |
| CD19+ B cell count, n/μL | 402.10±313.90 | 227.50±73.31 | 0.1675 |
| Response rate | 86.67% | 100.00% | 0.5109 |
| Prednisone dose before rituximab therapy, mg/day | 30.33±12.57 | 24.06±8.65 | 0.2232 |
| Prednisone dose at last follow up, mg/day | 6.67±7.24 | 5.00±8.02 | 0.6173 |
| Relapse rate before rituximab therapy, number/year | 1.6 | 1.15 | 0.2957 |
| Relapse rate after rituximab therapy, number/year | 0.05 | 0.19 | 0.2215 |
| Quantitative data was expressed as mean or mean ± standard deviation (SD), categorical data was presented as frequencies and percentages [n (%)]. Differences between catogorical data were analyzed by Fisher exact tests. Differences between quantitative data were analyzed by t-test. Abbreviations: eGFR, estimated glomerular filtration rate; MDRD, Modification of Diet in Renal Disease. *P < 0.05 for comparison of childhood onset group versus adult onset group. | | | |

| **Table-S4. Change of prednisone dosage after rituximab treatment** | | | | | | | | | |
| --- | --- | --- | --- | --- | --- | --- | --- | --- | --- |
|  | 0W | 3W | 6W | 12W | 20W | 32W | 44W | 56W | LFU |
| Prednisone, mg/day | 28.15 | 25.87 | 22.07 | 18.80 | 12.73 | 9.77 | 6.62 | 3.86 | 6.09 |
| Abbreviations: W, week; LFU, last follow up. | | | | | | | | | |

**Figures:**

### Figure-S1. | A: The variation of urine protein excretion of each patient after rituximab therapy; B: The variation of serum albumin of each patient after rituximab therapy; C: The variation of serum creatinine of each patient after rituximab therapy. Abbreviations: RTX, rituximab.

###
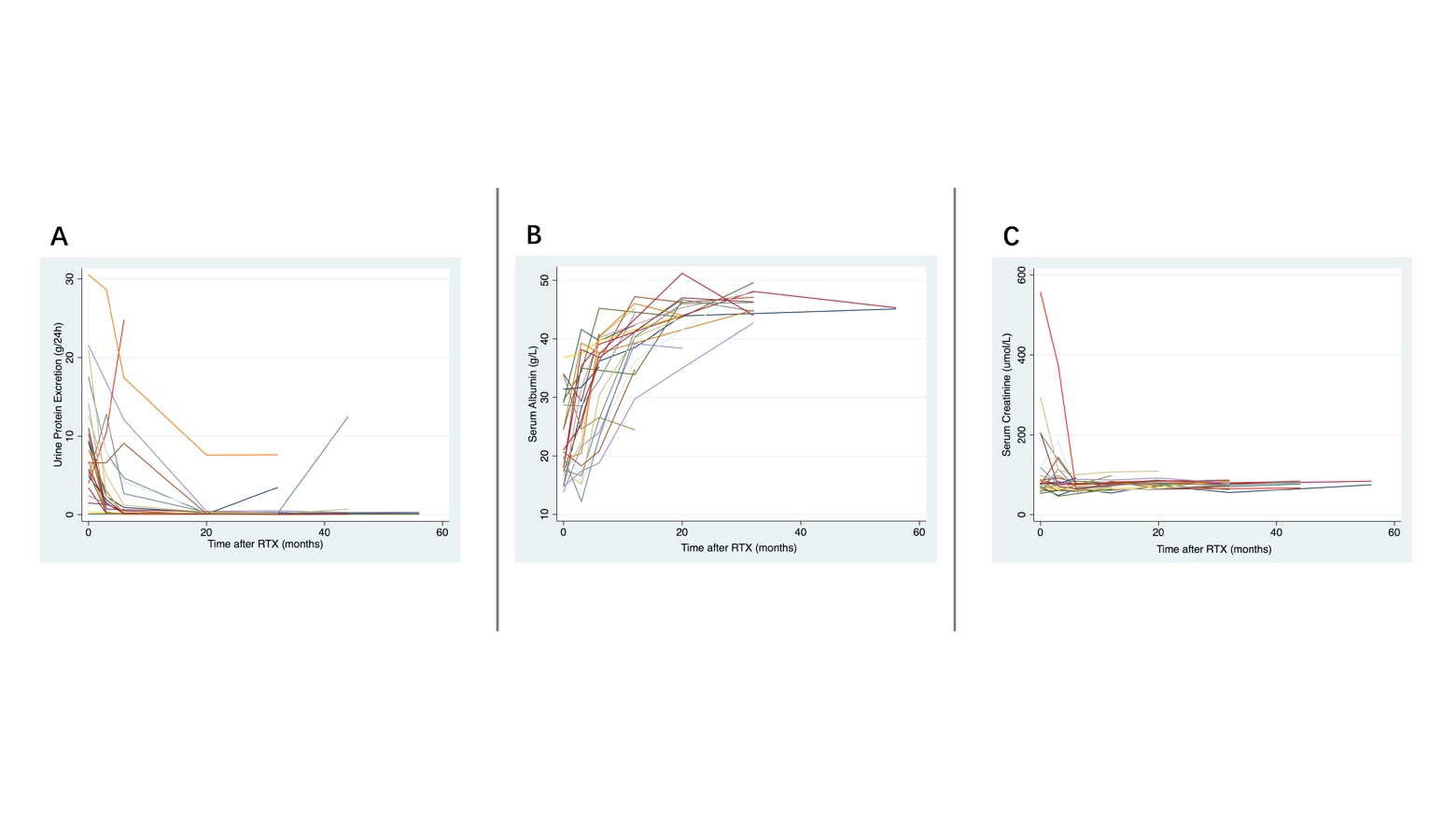

Supplement: Supplementary file 1 — Additional file 1: Table S1. Patient characteristics before rituximab therapy. Illustrating the age, immunosuppressive therapy, relapse rate, serum creatinine, serum albumin, urinary protein excretion, CD19+ B cell count of each patient before rituximab therapy. Showing rituximab courses and prednisone dosage of each participant receiving this therapy. Table S2. Baseline characteristics and outcome of rituximab therapy between patients with childhood-onset (<12y) and adult-onset MCD. Comparing the differences in the age at first rituximab infusion, 24h urinary protein excretion, serum creatinine, eGFR, serum albumin, CD19+ B cell count, response rate, prednisone dose before rituximab and at last follow up, relapse rate before and after rituximab between patients with childhood onset MCD (<12y) and patients with adult onset MCD (≥12y). Table S3. Baseline characteristics and outcome of rituximab therapy between patients with childhood-onset (<18y) and adult-onset MCD. Comparing the differences in the age at first rituximab infusion, 24h urinary protein excretion, serum creatinine, eGFR, serum albumin, CD19+ B cell count, response rate, prednisone dose before rituximab and at last follow up, relapse rate before and after rituximab between patients with childhood onset MCD (<18y) and patients with adult onset MCD (≥18y). Table S4. Change of prednisone dosage after rituximab treatment. Demonstrating the average dose of prednisone at each follow up time after rituximab therapy. Figure S1. A: The variation of urine protein excretion of each patient after rituximab therapy; B: The variation of serum albumin of each patient after rituximab therapy; C: The variation of serum creatinine of each patient after rituximab therapy. Abbreviations: RTX, rituximab. [file 12882_2021_2437_MOESM1_ESM.docx]
