# Supplementary material for: Extended infusion of rituximab combined with steroids is effective in inducing remission and reducing relapse in adult minimal change disease
Source: BMC Nephrol. 2021 Jul 1;22:242. doi: 10.1186/s12882-021-02437-4 (PMC8247102; doi:10.1186/s12882-021-02437-4)
Supplement: Supplementary file 2 — Additional file 2. Protocol of steroids tapering in the study. [file 12882_2021_2437_MOESM2_ESM.docx]

**Protocol of steroids tapering in the study:**

Tapering of prednisone started when partial or complete remission has been achieved. We tapered prednisone by 5 mg every 4 weeks (methylprednisolone by 4 mg every 4 weeks) when dosage of prednisone was over 15mg/d. Tapering was slowed down when dosage of prednisone was under 15mg/d by 5 mg every 8-12 weeks. For patients having higher dose of prednisone (over 30mg/d) at the beginning of therapy or having overt side effects of steroids, we accelerate the tapering of prednisone regardless of remission.
